# Retrospective cohort observation on psychotropic drug-drug interaction and identification utility from 3 databases: Drugs.com®, Lexicomp®, and Epocrates®

**DOI:** 10.1371/journal.pone.0287575

**Published:** 2023-06-22

**Authors:** Ravi Pinkoh, Ratchanee Rodsiri, Sorawit Wainipitapong

**Affiliations:** 1 Faculty of Pharmaceutical Sciences, Chulalongkorn University, Bangkok, Thailand; 2 Department of Pharmacology and Physiology, Faculty of Pharmaceutical Sciences, Chulalongkorn University, Bangkok, Thailand; 3 Institute of Psychiatry, Psychology, and Neuroscience, King’s College London, London, United Kingdom; 4 Department of Psychiatry and Center of Excellence in Transgender Health (CETH), Faculty of Medicine, Chulalongkorn University and King Chulalongkorn Memorial Hospital, the Thai Red Cross Society, Bangkok, Thailand; NHRI: National Health Research Institutes, TAIWAN

## Abstract

**Background:**

Pharmacotherapy is necessary for many people with psychiatric disorders and polypharmacy is common. The psychotropic drug-drug interaction (DDI) should be concerned and efficiently monitored by a proper instrument.

**Objectives:**

This study aimed to investigate the prevalence and associated factors of psychotropic DDI and to compare the identification utility from three databases: Drugs.com®, Lexicomp®, and Epocrates®.

**Methods:**

This was a retrospective cohort design. We collected demographic and clinical data of all patients hospitalised in the psychiatric inpatient unit in 2020. Psychotropic DDI profiles were examined through three databases. Descriptive statistics were used to report comprehensiveness of each database and prevalence of psychotropic DDI. The Fleiss’ kappa index would be analysed to indicate agreement strength of DDI severity classification among three databases.

**Results:**

From 149 total admissions, the psychotropic DDIs were found in 148 admissions (99.3%). Thorough the study, there were 182 of both psychotropic and other agents prescribed under 1,357 prescriptions. In total, 2,825 psychotropic DDIs were identified by using Drugs.com® 2,500 times, Epocrates® 2,269 times, and Lexicomp® 2,265 times. Interactions with clonazepam was the three most frequent agents when co-administrated with quetiapine (n = 56), risperidone (n = 36), and valproic acid and derivatives (n = 36). Serious DDIs were comparatively lower in incidence and there was no evidence of its association with reported clinical adverse consequences. The study revealed slight and fair agreement regarding severity classification among the three databases was found. DDI events detected by Drugs.com® were greatest in number, but Lexicomp® provided the broadest list of medications prescribed in our study.

**Conclusion:**

Among three databases, interactions detected by Drugs.com® were greatest in number, whereas Lexicomp® provided the broadest list of medications. Development of such databases, based on both theoretical and clinical conceptions, should be focused to balance safety of patients and weariness of healthcare providers.

## Background

Psychiatric disorders are among the most leading causes of burden globally and the number of patients with mental illnesses has been continuously increasing [1]. According to complex neurobiological aetiologies of several mental disorders, pharmacological treatments are commonly used and novel treatments, targeting on multidimensional of the pathophysiology, have been developed [2]. However, some patients who are unresponsive to standard treatment tend to be prescribed for multiple classes of psychotropic medication to reduce their symptoms [3]. Thus, the higher risks of psychotropic drug-drug interaction (DDI) are inevitable.

Apart from various psychiatric symptoms requiring different psychopharmacological actions, longer life expectancy, followed by further medical comorbidities, also warrants polypharmacy and increases risks of psychotropic DDIs [4]. Numerous previous studies have shown high prevalence of DDIs in various settings, either clinical or non-clinical, including nursing home (81.1%), community pharmacies (26.0–49.6%), inpatient and outpatient department of the hospital (22.3%) [5–7]. Despite the observed association between DDIs and clinical outcomes such as hospitalisation or hospital visits, only a few patients clinically manifested complications from DDIs [8, 9]. Nowadays, healthcare technology systems have been implemented to detect DDIs, but too frequent, false and unnecessary alarms can have negative effects on both providers’ exhaustion and patients’ safety [10]. Therefore, the multidisciplinary team should consider an appropriate instrument to identify clinically significant DDIs under the balance between alarm fatigue and patient safety.

Electronic databases used for DDI identification are widely used and varied in their sensitivity and comprehensiveness and those with sufficient information include Drugs.com®, Lexicomp®, and Epocrates® [11]. All mentioned databases have been used in the clinical practices and several previous studies have been carried out to investigate their DDIs identification utility in different countries, settings, and clinical populations [12–14]. However, identification utility of each database is diverse in terms of approved psychotropic medications or prescription patterns in individual countries [15]. Therefore, the drug list in each database, the number, and clinical significance of DDIs detected should be considered when selecting the appropriate database that balances the utilisation of efficient healthcare technology systems, as mentioned earlier. Regarding all aforementioned differences, studies examining databases for psychotropic DDI identification are indispensable.

Up to our knowledge, there have been no studies evaluating psychotropic DDIs from these three commonly used electronic databases (Drugs.com®, Lexicomp®, and Epocrates®) in the context of Thai hospital university setting. In addition to being frequently used in many healthcare systems, these three databases are comprehensive and suggest the management in case DDIs are detected. Some studies from Thailand also investigated DDI identification utility from two databases (Micromedex® and Drugs.com®) or three databases (Micromedex®, Drugs.com®, and Liverpool HIV interactions), but they did not identify psychotropic DDIs, and were not done in the real clinical situations [16, 17].

## Objectives

Our study primarily aimed to explore the prevalence and associated factors of psychotropic DDIs during a one-year observation in the psychiatric inpatient department of one Thai medical school. Secondly, we aimed to investigate psychotropic DDI identification utility from three databases e.g., Drugs.com®, Lexicomp®, and Epocrates®, based on the context of psychiatric hospitalisation in the quaternary hospital.

## Methods

This study was a retrospective cohort design. We extracted data from the electronic health records of all patients hospitalised during 2020 in the psychiatric inpatient unit at one university hospital in Bangkok, Thailand. The records included all pharmacological prescriptions which were recorded and followed from the first day of admission till the date of discharge or the end of our study. The psychotropic DDI was identified by three different databases. We analysed the association between psychotropic DDI and clinical outcomes, including recorded adverse drug events, length of hospitalisation, and readmission. The informed consent was not obtained because we collected data from service records and report anonymously. All patients with age over than 18 years old were included, thus neither sample size calculation nor sampling was done in this study. This study was granted the ethical approval from the Institutional Review Board of Faculty of Medicine, Chulalongkorn University.

### Demographic and clinical data

Demographic and clinical data, including age, gender, psychiatric and medical diagnoses, were recorded. All prescriptions during the entire hospitalisation were extracted for each prescribed agent, dosage, and date of prescription. Thorough the admission, adverse drug reaction and length of hospitalisation were noted by their attending physicians. A history of readmission within one year after discharge was also collected.

### Psychotropic drug-drug interaction

Drugs prescribed simultaneously during hospitalisation and found to potentially develop DDI from each database would be reported as an event of DDI. With three databases used in our study, all DDIs were detected, categorised them according to severity and reported separately. All included databases can be accessed publicly or under subscription and often used in our country. However, none of them is considered a standard database regarding the policy of neither national nor institutional level. Our focus was on drug-drug interactions, which included interactions with nutritional supplements. However, we excluded interactions involving alcohol, smoking, and illicit substances from our study as they were beyond the scope of our objectives and were not permitted during the hospitalisation period.

### Drugs.com®

Drugs.com® is an independent medicine information website, aiming to be a comprehensive resource for both healthcare professionals and consumers. Its database is powered by several organisations and peer-reviewed. Three levels of DDI severity are classified and listed below [18].

*Major* Highly clinically significant and avoid combinations; the risk of DDI outweighs the benefit
*Moderate* Moderately clinically significant and usually avoid combinations; use only under special circumstances
*Minor* Minimally clinically significant; minimise risk, assess risk and consider an alternative drug
*Unknown* No DDI information available

### Lexicomp®

Lexicomp® is a DDI database for clinicians and needs subscription for access. It has been developed in various platforms, such as mobile applications, online, or desktop software. The risks of DDI are rated from A (no known interaction) to X (avoid combination) [19].

*A* No known interaction, neither PK nor PD DDI is demonstrated
*B* No action needed, little to no evidence of clinical concern
*C* Monitor therapy, clinically significant but benefit usually outweighs risk, dosage adjustment may be needed
*D* Consider therapy modification, patient-specific assessment, aggressive monitoring, empiric dosage changes, or alternative agents
*X* Avoid combination, clinically significant and risk outweighs benefit, and generally considered contraindicated

### Epocrates®

Epocrates® is a database used for not only DDI checking but also several healthcare services such as pill identification, medical calculator, and information resources for patients. The severity of DDI is stratified into four levels, including *Contraindicated*, *Avoid/Use Alternative*, *Monitor/Modify Therapy*, and *Caution Advised* [20].

### Statistical analysis

The SPSS version 29.0 (IBM) was utilised in this study. Descriptive statistics were used to report the demographic data. Categorical variables were presented as counts and percentage, while continuous variables were described using the mean ± standard deviation or the median [interquartile range] depending on data distribution. The Fleiss’ kappa index would be analysed to indicate agreement strength of DDI severity classification among three databases with value of 0.81–1.00 (almost perfect), 0.61–0.80 (substantial), 0.41–0.60 (moderate), 0.21–0.40 (fair), 0.00–0.20 (slight), or below 0.00 (poor). Pearson or Spearman correlation would be analysed appropriately to determine the correlation between numbers of psychotropic DDIs and associated variables. All tests were two-tailed and a P-value of ≤ 0.05 was considered statistically significant.

## Results

Throughout the study, there were 149 psychiatric admissions from 128 patients. Most patients were female (n = 75, 58.6%), diagnosed with schizophrenia spectrum disorders (n = 42, 32.8%), and averagely at 39.4 ± 18.8 years of age (min–max = 19–86 years old). All 1,357 prescriptions during the study period were assessed. In total, 176 individual agents of either psychotropic or other medications were prescribed. Three most frequently prescribed psychotropic agents were clonazepam (n = 109, 8.0%), quetiapine (n = 82, 6.0%), and haloperidol (n = 64, 4.7%). Seven patients (5.5%) reported history of adverse drug reactions and sixteen patients (12.5%) were readmitted within a year. The demographic, clinical, and prescription data of all patients are shown in **Table 1**.

**Table 1:** Demographic, clinical, and prescription data of all participants.

Demographic and Clinical data (N = 128)	N (%) Mean ± S.D. Median [IQR]	Psychotropic Medication Prescription data (N = 1357)	N (%)
Sex—Female	75 (58.6%)	Antipsychotics—Typical Atypical	104 (7.7%) 248 (18.3%)
Age (years)	39.4 ± 18.8		
Primary Psychiatric Diagnosis • Schizophrenia spectrum disorders • Major depressive disorder • Bipolar and related disorders • Organic mental disorders • Neurocognitive disorders • Others	45 (36.7%) 33 (25.7%) 22 (17.2%) 6 (4.6%) 4 (3.1%) 16 (12.5%)	Antidepressant—SSRIs SNRIs SARI TCAs and NaSSAs Others	49 (3.6%) 27 (2.0%) 50 (3.7%) 17 (1.3%) 14 (1.0%)
		Antimanic -Antiepileptics Lithium	94 (6.9%) 16 (1.2%)
Presence of medical comorbidities	63 (49.2%)	Benzodiazepines and hypnotics	243 (17.9%)
Readmission within 1 year	16 (12.5%)	Anticholinergics—Trihexyphenidyl	64 (4.7%)
Length of hospitalisation (days)	24 [14,38]	Cognitive enhancers	4 (0.3%)
Total number of medications prescribed	9 [6,12]	Others psychotropic agents	12 (0.9%)
**Non-Psychotropic Medication Prescription data (N = 1357)**			
**Classification**	**N (%)**	**Classification**	**N (%)**
Antimicrobial or antiviral agents	11 (0.8%)	Antihypertensive agents	59 (4.3%)
Antacid or Proton-pump inhibitors	20 (1.5%)	Antidiabetic agents	27 (2.0%)
Anti-allergic agents	17 (1.3%)	Hypolipidemic agents	17 (3.3%)
Anti-pyretic agents (e.g., paracetamol)	81 (6.0%)	Anti-coagulant agents	8 (0.6%)
Vitamins, minerals, supplements	51 (3.8%)	Others	124 (9.1%)

Only 1 out of 149 (99.3%) admissions had no psychotropic DDI during hospitalisation. In total, 956 DDI patterns with 2,825 pairs of psychotropic DDIs were identified based on all databases. According to Drugs.com®, Lexicomp®, and Epocrates®, DDIs were identified 2,500 times, 2,269 times, and 2,265 times, respectively. There were 26 and 17 serious psychotropic DDIs identified by Lexicomp® (X category) and Epocrates® (Contraindicated category); meanwhile, 254 DDIs were classified as Major category from Drugs.com®. Three most frequent DDIs were events of co-administrated clonazepam with quetiapine (n = 56), risperidone (n = 36), and valproic acid and derivatives (n = 36). **Table 2** displays the top 20 frequent agents causing psychotropic DDI and pairs of psychotropic DDI, as well as their severity regarding different databases. The serious DDIs found from our study are shown in **Table 3**.

**Table 2:** The top 20 frequent agents causing DDI, pairs of psychotropic DDI and their severity.

Agents causing DDI (N = 956)	N (%)	Pairs of Psychotropic DDI (N = 2,825)	N (%)	Psychotropic DDI Severity		
				Drugs.com®	Lexicomp®	Epocrates®
Quetiapine	97 (10.1%)	Clonazepam—Quetiapine	56 (2.0%)	Moderate	C	Caution^b^
Clonazepam	83 (8.7%)	Clonazepam—Risperidone	36 (1.3%)	Moderate	C	Caution^b^
Trazodone	78 (8.2%)	Clonazepam—Valproic^a^	36 (1.3%)	Moderate	C	Monitor^c^
Risperidone	69 (7.2%)	Risperidone—Trihexyphenidyl	36 (1.3%)	Moderate	C	Caution^b^
Aripiprazole	62 (6.5%)	Clonazepam—Trihexyphenidyl	32 (1.1%)	Moderate	-	Caution^b^
Olanzapine	57 (6.0%)	Quetiapine—Valproic^a^	30 (1.1%)	Moderate	C	Monitor^c^
Sertraline	52 (5.4%)	Clonazepam - Trazodone	29 (1.0%)	Moderate	C	Monitor^c^
Trihexyphenidyl	51 (5.3%)	Aripiprazole - Clonazepam	28 (1.0%)	Moderate	C	Monitor^c^
Lorazepam	47 (4.9%)	Quetiapine - Trihexyphenidyl	28 (1.0%)	Moderate	C	Caution^b^
Zolpidem	46 (4.8%)	Quetiapine - Trazodone	28 (1.0%)	Moderate	C	Monitor^c^
Diazepam	44 (4.6%)	Trihexyphenidyl - Valproic^a^	28 (1.0%)	Moderate	C	Monitor^c^
Valproic^a^	44 (4.6%)	Quetiapine - Risperidone	28 (1.0%)	Moderate	C	Monitor^c^
Paliperidone	44 (4.6%)	Risperidone - Valproic^a^	23 (0.8%)	Moderate	C	Monitor^c^
Haloperidol	43 (4.5%)	Haloperidol - Trihexyphenidyl	22 (0.8%)	Moderate	B	Monitor^c^
Mirtazapine	40 (4.2%)	Clonazepam - Haloperidol	19 (0.7%)	Moderate	C	Monitor^c^
Escitalopram	39 (4.1%)	Haloperidol - Risperidone	19 (0.7%)	Major	C	Monitor^c^
Venlafaxine	38 (4.0%)	Lorazepam - Quetiapine	19 (0.7%)	Moderate	C	Monitor^c^
Clozapine	36 (3.8%)	Risperidone - Trazodone	19 (0.7%)	Moderate	C	Monitor^c^
Propranolol	34 (3.6%)	Clonazepam—Venlafaxine	15 (0.5%)	Moderate	-	Monitor^c^
Perphenazine	34 (3.6%)	Clonazepam - Sertraline	15 (0.5%)	Moderate	C	Monitor^c^

*DDI*–Drug-drug interaction

*a* Valproic Acid and Derivatives

*b* Caution advised

*c* Monitor / Modify therapy

**Table 3:** The serious DDIs found from our study.

DDIs	Mechanism or clinical effects	Lexicomp	Drugs.com	Epocrates
APZ—ORD	CNS depression (Synergistic effect)	X	Moderate (monitor)	Caution Advised
CLZ—ORD	CNS depression (Synergistic effect)	X	Moderate (monitor)	Monitor / Modify therapy
FLZ—FPH	CNS depression (Additional effect)	X	-	-
FLZ—HAL	CNS depression (Additional effect)	X	-	-
LMG—ORD	CNS depression (Synergistic effect)	X	Moderate (monitor)	Caution advised
PGL—ORD	CNS depression (Synergistic effect)	X	Moderate (monitor)	Caution advised
QTP—ORD	CNS depression (Synergistic effect)	X	Moderate (monitor)	Caution advised
TRA—ORD	CNS depression (Synergistic effect)	X	Moderate (monitor)	Caution advised
VPA—ORD	CNS depression (Synergistic effect)	X	Moderate (monitor)	Caution advised
GEM—SMV	GEM inhibit OATP1B1 and CYP2C8 and ↑ risk rhabdomyolysis of SMV	X	Major (contraindicated)	Contraindicated
BUP—VTX	↑ VTX plasma level by CYP2D6 inhibition	D	Major (adjust dose)	Avoid / Use alternative
LMG—VPA	↑ LMG plasma level by inhibiting glucuronidation	D	Major (adjust dose)	Monitor / Modify therapy
MIRT—VFX	5HT-1A and 2A receptor stimulation and cause serotonin syndrome	C	Major (generally avoid)	Avoid / Use alternative
CLZ—OLZ	CNS depression (Additional effect)	C	Major (generally avoid)	Monitor / Modify therapy

*DDI*–Drug-drug Interaction; *CNS*–Central nervous system; *CYP*–Cytochrome P; *APZ*–Aripiprazole; *ORD*–Orphenadrine; *CLZ*–Clonazepam; *FLZ*–Flunarizine; *FPH*–Fluphenazine; *HAL*–Haloperidol; *LMG*–Lamotrigine; *PGL*–Pregabalin; *QTP*–Quetiapine; *TRA*–Trazodone; *VPA*–Valproic acid and derivatives; *GEM*–Gemfibrozil; *SMV*–Simvastatin; *BUP*–Bupropion; *VTX*–Vortioxetine; *MIRT*–Mirtazapine; *VFX*–Venlafaxine; *OLZ*–Olanzapine

The Spearman’s correlation between numbers of identified psychotropic DDI and associated variables is demonstrated in **Table 4**. There was a very strong positive correlation between numbers of total medications prescribed during admission and numbers of psychotropic DDI (Spearman’s rho = 0.827, P-value < 0.001). Meanwhile, a moderate positive correlation between numbers of DDI and readmission within 1 year (Spearman’s rho = 0.391, P-value < 0.001), length of hospitalisation (Spearman’s rho = 0.374, P-value < 0.001), and having underlying diseases (Spearman’s rho = 0.331, P-value < 0.001) was found. Our results also suggested a weak positive correlation between age and numbers of psychotropic DDI (Spearman’s rho = 0.286, P-value = 0.002). According to prescription profiles, both antihypertensive and antiepileptic agents were strongly correlated with DDI (Spearman’s rho = 0.468, P-value < 0.001 and Spearman’s rho = 0.416, P-value < 0.001). We also found a weak correlation between DDIs and either benzodiazepines (Spearman’s rho = 0.220, P-value = 0.016) or other supportive and psychiatric agents (Spearman’s rho = 0.277, P-value = 0.002 and Spearman’s rho = 0.272, P-value = 0.003).

**Table 4:** Correlation between numbers of identified DDI and associated variables.

Variables	Spearman’s rho	P-value	Variables	Spearman’s rho	P-value
Age	0.286	0.002*	Number of total med^a^	0.827	< 0.001*
Gender—male	-0.119	0.198	Length of hospitalisation	0.374	< 0.001*
Underlying diseases	0.331	< 0.001*	Readmission with 1 year	0.391	< 0.001*
Antipsychotic—typical	0.160	0.083	Antidepressant	0.080	0.387
Antipsychotic—atypical	0.112	0.223	Benzodiazepine & hypnotic	0.220	0.016*
Antimanic—antiepileptic	0.416	< 0.001*	Trihexyphenidyl	0.108	0.240
Antimanic—lithium	0.122	0.186	Other psychiatric agents	0.272	0.003*
Antihypertensive	0.468	< 0.001*	Other supportive agents	0.277	0.002*

*a* medication prescribed

* P-value ≤ 0.05

**Table 5** demonstrates the kappa index of agreement strength of psychotropic DDI severity classification among three databases. According to Fleiss’ kappa index, the overall kappa was 0.162 (95% CI 0.14–0.19, P-value < 0.001). Meanwhile kappa index of Drugs.com® - Lexicomp® was 0.04 (P-value = 0.07), Drugs.com® - Epocrates® was 0.21 (P-value < 0.001), and Lexicomp® - Epocrates® was 0.24 (P-value < 0.001).

**Table 5:** Agreement of psychotropic DDI severity classification among three databases.

Severity classification	Drugs.com®	Lexicomp®	Epocrates®	Kappa (95% CI)	P-value	Strength
Minor	Minor	B	Caution^a^	0.02 (-0.02–0.05)	0.33	Slight
Moderate	Moderate	C	Monitor^b^	0.14 (0.10–0.18)	<0.001*	Slight
Major	Major	D	Avoid^c^	0.23 (0.20–0.27)	<0.001*	Fair
Contraindicated	N/A	X	Contra^d^	0.08 (0.04–0.11)	<0.001*	Slight

*a* Caution advised

*b* Monitor / Modify Therapy

*c* Avoid / Use Alternative

*d* Contraindicated

* P-value ≤ 0.05

## Discussion

The psychotropic DDI occurred in nearly all psychiatric admissions. Interestingly, the only admission without psychotropic DDI was a patient admitted for drug dependence rehabilitation and prescribed for vitamin supplements and supportive therapies. Our findings confirmed the high prevalence of psychotropic DDIs detected by electronic databases in the psychiatric inpatient settings [21, 22].

However, the median of number of medications prescribed during hospitalisation was 9 and this limited its generalisability with patients in the psychiatric outpatient department, where the number of medications prescribed was comparatively lower [23]. Due to the nature of quaternary hospital services, patients in our institute often had more complicated medical comorbidities and then were at higher risk for psychotropic DDIs.

Among all patterns of psychotropic DDI pairs, the top-three psychotropic agents causing DDIs (i.e., quetiapine, clonazepam, and trazodone) were used to remedy sleep disturbances. Apart from its indication for both psychotic and affective disorders, quetiapine has been widely prescribed for sleep problems, even it is an off-label use, because of its lower addictive qualities compared to benzodiazepines [24]. Similarly, either clonazepam or trazodone are known for their sleep disorders management, especially for trazodone, in older patients or patients with depression [25, 26]. Yet, several adverse reactions from these medications alone or their DDIs could be found. There were only 6 out of 149 admissions that clinical adverse reactions were recorded. Most of them were explained by an idiosyncratic agent except one patient with schizophrenia and prescribed olanzapine and clonazepam. Additionally administrated trazodone, the patient had developed retrograde amnesia afterwards, which could be caused by DDI of trazodone and such medications. During psychiatric hospitalisation, implementation of insomnia psychosocial interventions will therefore be advantageous in lowering both sedative agents prescription and incidence of psychotropic DDIs [27].

Correlation between higher numbers of psychotropic DDIs and several associated factors was found. It was not surprise that patients with greater numbers of medications prescribed during hospitalisation would be strongly correlated with more events of psychotropic DDIs. Weak correlation with ageing and presence of medical comorbidities was also observed as these groups were at risk of polypharmacy [28]. Because numbers of psychotropic DDI events were cumulatively recorded, correlation with DDIs was found in patients with longer hospitalisation or readmission.

We found a moderate correlation between DDIs and two medication classes: antiepileptic and antihypertensive agents. Antiepileptic agents are known for their frequent DDIs with other psychotropic medications under various mechanisms, especially through pharmacodynamic interaction [29]. Many psychotropic medications are related with orthostatic hypotension, so psychotropic DDIs will be identified when co-prescribing with antihypertensive agents [30]. Also, additive effects on sedation could be caused by co-administration of benzodiazepines and hypnotics [31]. To prevent adverse psychotropic DDIs, clinicians should be considerate prior to prescribing such groups of medications.

Our findings found the discrepancy between numbers of identified psychotropic DDIs and reports of clinical adverse reactions, which was consistent with previous studies [32]. The severity of the identified DDIs may not always be high enough to cause a clinically significant adverse reaction. Additionally, the identified DDIs might be managed appropriately by healthcare providers such as adjusting dosages. It also emphasised the importance of DDI detection alert system that needed both clinical and informational integration [33].

According to the number of detected psychotropic DDIs, Drugs.com® was a comparatively more sensitive instrument. Compared to Epocrates® and Lexicomp®, more than 130 times of psychotropic DDIs were detected from Drugs.com® but none of clinically significant events was shown. The agreement strength among three databases’ severity classification was slight and Lexicomp® - Epocrates® had the strongest agreement. Even though both Lexicomp® and Epocrates® comprise 4 severity classifications, Lexicomp® database provide most extensive list of medications for our study as it included some medications approved in Thailand but disapproved in other countries, such as domperidone or flunarizine. Information regarding drug interaction with herbal medicine was also given, and we considered, from three examined databases, that Lexicomp® held the most suitability for its psychotropic DDI identification utility in the context of our country.

Interestingly, identified DDIs classified as major severity were found in DDIs caused by both non-psychotropic drugs (gemfibrozil-simvastatin and omeprazole-rilpivirine) or by psychotropic and non-psychotropic drug interaction (flunarizine and orphenadrine). None of patients receiving mentioned pairs of DDIs reported any clinical adverse reaction. Meanwhile, psychotropic DDIs from dual psychotropic agents were much more common and classified as less severe categories. In psychiatric practices, several identified pairs of psychotropic DDIs were broadly prescribed, such as antipsychotic agents and trihexyphenidyl, as observed in our study [23]. Despite the fact that they were less clinically significant, co-administration of such medications should be carefully considered by clinicians in terms of benefits and risks of negative consequences. Furthermore, considerate clinical judgment should be highly concerned when co-prescribing psychotropic agents with other non-psychotropic medications, particularly flunarizine and orphenadrine, as found in our study.

Our study investigated psychotropic DDIs occurring within one-year long in the clinical setting, so it reflected the real situation of psychotropic DDIs in the psychiatric inpatient department of the quaternary hospital where several non-psychotropic agents were co-administrated. All examined databases are commonly used and comparative studies of them are still lacking. Some limitations should be addressed. We retrospectively extracted data from the psychiatric record which some information or associated factors might be missed or not enrolled in case of unrecorded. Some prescriptions with severe DDI might be warned by the hospital and such prescriptions were prohibited. Psychiatric diagnoses were mainly schizophrenia spectrum and major depressive disorders, so psychotropic medications out of these two indications were less prescribed and analysed in our study. The rate of clinical adverse reactions was low and the association with DDIs could not be well identified. As mentioned earlier, our study was conducted in the quaternary hospital, so the results might not be generalised to other types of healthcare settings or to populations with different characteristics. However, implications of the results still applied to non-mental hospitals where patients were supposed to be prescribed both medication classes, reported in our study.

Further studies should be conducted to develop updated databases for psychotropic DDI identification in specific populations. The comparison of DDIs among databases in terms of drug mechanisms would enable professionals to select the most suitable database and be helpful for clinicians’ practical implications. Apart from theoretical interactions, clinical significance should be focused and appropriately alerted to prevent alarm fatigue. However, it is challenging to integrate both clinical and informational databases as psychotropic DDIs are tremendously common, and their serious interactions are also difficult to be predicted.

## Conclusion

Almost all patients hospitalised in the psychiatric inpatient unit had at least one psychotropic DDI, but clinical adverse consequences were rarely reported. Among three databases, interactions detected by Drugs.com® were greatest in number, whereas Lexicomp® provided the broadest list of medications. Development of such databases based on both theoretical and clinical conceptions should be focused to balance safety of patients and weariness of healthcare providers.
